# Mechanisms of BK Channel Activation by Docosahexaenoic Acid in Rat Coronary Arterial Smooth Muscle Cells

**DOI:** 10.3389/fphar.2018.00223

**Published:** 2018-03-27

**Authors:** Ling-Ling Qian, Man-Qing Sun, Ru-Xing Wang, Tong Lu, Ying Wu, Shi-Peng Dang, Xu Tang, Yuan Ji, Xiao-Yu Liu, Xiao-Xi Zhao, Wen Wang, Qiang Chai, Min Pan, Fu Yi, Dai-Min Zhang, Hon-Chi Lee

**Affiliations:** ^1^Department of Cardiology, Wuxi People’s Hospital Affiliated to Nanjing Medical University Wuxi, China; ^2^Department of Cardiovascular Medicine, Mayo Clinic, Rochester, MN, United States; ^3^State Key Laboratory of Pharmaceutical Biotechnology, School of Life Sciences, Nanjing University, Nanjing, China; ^4^Department of Physiology, Institute of Basic Medicine, Shandong Academy of Medical Sciences, Jinan, China; ^5^Department of Cardiology, Affiliated Hospital Nantong University, Nantong, China; ^6^Department of Cardiovascular Diseases, Xijing Hospital, Fourth Military Medical University, Xi’an, China; ^7^Nanjing First Hospital, Nanjing, China

**Keywords:** docosahexaenoic acid, BK channel, cytosolic calcium, PLC–IP_3_ signal pathway, coronary arterial smooth muscle cells

## Abstract

**Aim:** Docosahexaenoic acid (DHA) is known to activate the vascular large-conductance calcium-activated potassium (BK) channels and has protective effects on the cardiovascular system. However, the underlying mechanisms through which DHA activates BK channels remain unclear. In this study, we determined such mechanisms by examining the effects of different concentrations of DHA on BK channels in freshly isolated rat coronary arterial smooth muscle cells (CASMCs) using patch clamp techniques.

**Methods and Results:** We found that BK channels are the major potassium currents activated by DHA in rat CASMCs and the effects of DHA on BK channels are concentration dependent with a bimodal distribution. At concentrations of <1 μM, DHA activated whole-cell BK currents with an EC_50_ of 0.24 ± 0.05 μM and the activation effects were abolished by pre-incubation with SKF525A (10 μM), a cytochrome P450 (CYP) epoxygenase inhibitor, suggesting the role of DHA-epoxide. High concentrations of DHA (1–10 μM) activated whole-cell BK currents with an EC_50_ of 2.38 ± 0.22 μM and the activation effects were unaltered by pre-incubation with SKF525A. Single channel studies showed that the open probabilities of BK channels were unchanged in the presence of low concentrations of DHA, while significantly increased with high concentrations of DHA. In addition, DHA induced a dose-dependent increase in cytosolic calcium concentrations with an EC_50_ of 0.037 ± 0.01 μM via phospholipase C (PLC)–inositol triphosphate (IP_3_)–Ca^2+^ signal pathway, and inhibition of this pathway reduced DHA-induced BK activation.

**Conclusion:** These results suggest that DHA can activate BK channels by multiple mechanisms. Low concentration DHA-induced BK channel activation is mediated through CYP epoxygenase metabolites, while high concentration DHA can directly activate BK channels. In addition, DHA at low and high concentrations can both activate BK channels by elevated cytosolic calcium through the PLC–IP_3_–Ca^2+^ signal pathway.

## Introduction

Long-chain *n*-3 polyunsaturated fatty acids, which mainly include docosahexaenoic acid (DHA) with a 22-carbon chain and eicosapentaenoic acid (EPA) with a 20-carbon chain, play a critical role in protecting cardiovascular function (Ramel et al., 2010; Saravanan et al., 2010; Liu et al., 2011; Colussi et al., 2014). Both EPA and DHA have been shown to be metabolized by cytochrome P450 (CYP) epoxygenase (Agbor et al., 2012). DHA has been shown to reduce adverse cardiovascular events through arrhythmia suppression, vasodilatation, increased coronary artery blood flow, and lowering blood pressure (Ramel et al., 2010; Saravanan et al., 2010; Liu et al., 2011; Colussi et al., 2014). In addition, DHA is known to have the properties of reducing platelet aggregation, anti-inflammation, lowering plasma triglyceride, and modulation of ion channel function (Breslow, 2006; Lavie et al., 2009; Oh et al., 2010; Yan et al., 2013).

There are at least five types of potassium (K^+^) channels in coronary arterial smooth muscle cells (CASMCs): the voltage-gated potassium (Kv) channels, the ATP-sensitive K^+^ (K_ATP_) channels, the large-conductance (BK), the intermediate-conductance (IK), and the small-conductance (SK) calcium (Ca^2+^)-activated K^+^ channels (Wang et al., 2011). Among these K^+^ channels, the BK channels underlie the predominant ionic mechanism that regulates membrane potentials of vascular smooth muscle cells which in turn controls vascular tone (Horrigan and Aldrich, 2002; Hoshi et al., 2013a). BK channel function is frequently altered in various physiological and pathophysiological conditions, including hypertension, shock, diabetes, and ischemic heart disease (Hu and Zhang, 2012). Therefore, the BK channel may be a potential therapeutic target for the treatment of cardiovascular diseases. Previous studies have shown that coronary arterial dysfunction is associated with impaired BK channels, especially in diabetes mellitus and hypertension (Lu et al., 2012; Hoshi et al., 2013c; Yi et al., 2014). It has also been reported that DHA binds with high affinity to BK channels, resulting in lowering of blood pressure (Hoshi et al., 2013b,c). We have also shown that DHA can activate vascular BK channels, leading to coronary artery vasodilatation (Wang et al., 2011). In our previous report, we have found that activation of BK channels by DHA at concentrations of <1 μM is dependent on the CYP epoxygenase activity (Wang et al., 2011). In this study, we investigated the mechanisms of BK channel activation by DHA other than that dependent on CYP epoxygenase activity.

In the present study, we found that the profile of BK channel activation by DHA has a bimodal distribution. We therefore hypothesized that the mechanisms of BK channel activation by DHA are different at low and high concentrations. To test this hypothesis, we determined the mechanisms of BK channel activation by DHA at different concentrations using patch clamp techniques and cytosolic calcium fluorescent ratio measurements. We found that activation of BK channels by DHA at concentrations >1 μM is independent of CYP epoxygenase activity. In addition, DHA at low and high concentrations can both activate BK channels by elevated cytosolic calcium via the phospholipase C (PLC)–inositol triphosphate (IP_3_)–Ca^2+^ signal pathway. These findings indicate that DHA activates BK channels through multiple mechanisms.

## Materials and Methods

### Experimental Animals

Eight-week-old male Sprague-Dawley rats (250–300 g) were obtained from the Harlan Laboratories (Madison, WI, United States) and from Shanghai SLAC Laboratory Animal Co., Ltd. (Shanghai, China). This study was carried out in accordance with the recommendations of the Guide for the Care and Use of Laboratory Animals published by the US National Institutes of Health (2011). The protocol was approved by the Institutional Animal Care and Use Committee of Mayo Foundation and the Committee on Animal Care of Wuxi People’s Hospital affiliated to Nanjing Medical University.

### Isolation of Rat Coronary Arterial Smooth Muscle Cells

Rat CASMCs were dissociated enzymatically, as described previously (Wang et al., 2012). Briefly, rats were anesthetized with isoflurane (2%), then rat hearts were rapidly excised and then placed in cold (4°C) physiological saline solution (in millimolar): NaCl 145.0, KCl 4.0, CaCl_2_ 0.05, MgCl_2_ 1.0, 4-(2-hydroxyethyl)-1-piperazineethanesulfonic acid (HEPES) 10.0, and glucose 10.0 at pH 7.4 with NaOH. Rat coronary arteries (the septal, right and left anterior descending coronary arteries) were carefully dissected free of surrounding myocardium and connective tissue. Freshly isolated rat coronary arteries were incubated with 1.0 ml physiological saline solution containing bovine serum albumin (0.1%, w/v) for 10 min at 37°C in a shaking water bath followed by digestion in 1.0 ml saline solution containing 1.5 mg papain, 1.0 mg dithiothreitol, and 0.1% bovine serum albumin at 37°C for 10 min. The vessels were further treated with 1.0 ml saline solution containing 1.0 mg collagenase, 1.0 mg trypsin inhibitor, 0.25 mg elastase, and 0.1% bovine serum albumin at 37°C for another 10 min and then washed three times with 1.0 ml aliquots of saline solution. The vessels were gently triturated with a fire-polished glass pipette until cells were completely dissociated.

### Whole-Cell and Single Channel Electrophysiology

Whole-cell and inside-out single BK currents were recorded from freshly isolated rat CASMCs as we have previously described (Wang et al., 2011) using an Axopatch 200B Amplifier (Molecular Devices, Inc., Sunnyvale, CA, United States), filtered at 2 kHz, and sampled at 50 kHz. All experiments were performed at room temperature (22–24°C). To investigate the effects of different concentrations of DHA on BK currents, DHA was perfused cumulatively from 0.01 to 10 μM on one cell. Whole-cell K^+^ currents were recorded from a holding potential (HP) of -60 mV with pulses of 100 ms duration to testing potentials (TPs) from -40 to +160 mV in 10 mV increments. Pipette resistance was 2.0–4.0 MΩ when filled with the pipette solution which contained (in millimolar) KCl 140.0, MgCl_2_ 0.5, Na_2_ATP 5.0, Na_2_GTP 0.5, HEPES 10.0, ethylene glycol tetraacetic acid (EGTA) 1.0, CaCl_2_ 0.465 (∼200 nM free Ca^2+^) at pH 7.35. The bath solution for whole-cell recordings contained (in millimolar) NaCl 145.0, KCl 5.6, MgCl_2_ 1.0, CaCl_2_ 0.5, HEPES 10.0, and glucose 10.0 at pH 7.4. To investigate the activation effects of DHA on different K^+^ channels, various K^+^ channel blockers were applied after baseline K^+^ current recordings were obtained. In brief, we used tetraethylammonium (TEA, 10 mM) for total K^+^ currents, iberiotoxin (IBTX, 100 nM) for BK channels, 1-[(2-chlorophenyl)diphenylmethyl]-1H-pyrazole (TRAM-34, 200 nM) for IK channels, apamin (APA, 1 μM) for SK channels, 4-aminopyridine (4AP, 5 mM) for Kv channels, and glyburide (GLY, 10 μM) for K_ATP_ channels.

Inside-out single BK channel currents were elicited at +60 mV. The BK channels were identified by their unitary conductance, voltage sensitivity, and Ca^2+^ sensitivity. Channel open probabilities (NPO) were calculated using Clampfit 10.2 software (Axon Instruments, Foster City, CA, United States). Pipette resistance was 5–10 MΩ when filled with the pipette solution which contained (in millimolar) KCl 140, CaCl_2_ 1, MgCl_2_ 1, HEPES 10, and EGTA 1 at pH 7.4. The bath solution contained (in millimolar) KCl 140, MgCl_2_ 1, EGTA 1, HEPES 10, CaCl_2_ 0.816 (1 μM free Ca^2+^) at pH 7.35.

### Cytosolic Calcium Concentration Measurements

Cytosolic calcium concentrations in CASMCs were measured by LAMBDA DG-4 (Sutter Company, United States) as we have previously reported (Wang et al., 2012). Briefly, the acutely isolated normal rat CASMCs were incubated protected from light with 5 μM Fura-2/AM, a fluorescent dye, in calcium-free Hanks’ solution containing NaCl 137.93, KCl 5.33, Na_2_HPO_4_•12H_2_O 0.363, KH_2_PO_4_ 0.441, and NaHCO_3_ 4.17 (in millimolar) for 30 min. The cells were then washed and superfused with Hanks’ solution in a perfusion bath mounted on the stage of an inverted microscope. Fluorescence intensities were recorded with excitation wavelengths 340 and 380 nm, and emission wavelength 510 nm. Fluorescent intensity ratios of F340/F380 were calculated, and the changes of fluorescent intensity ratios were used to detect the changes of cytosolic calcium concentrations in this study as previously reported (Paudel et al., 2014).

### Chemicals

All chemicals were obtained from Sigma-Aldrich Co. (St. Louis, MO, United States), except DHA was purchased from Cayman Chemical (Ann Arbor, MI, United States) and SKF525A from BIOMOL (Plymouth Meeting, PA, United States). In this study, we defined low concentrations of DHA as <1 μM and high concentrations as >1 μM.

### Statistical Analysis

Data were presented as mean ± SEM. Student’s *t*-test was used to compare data between two groups. One-way ANOVA followed by contrast testing was used to compare data from multiple groups. Statistical significance was defined as *P* < 0.05.

## Results

### Effects of DHA on Different K^+^ Currents in Rat CASMCs

**FIGURE 1:**
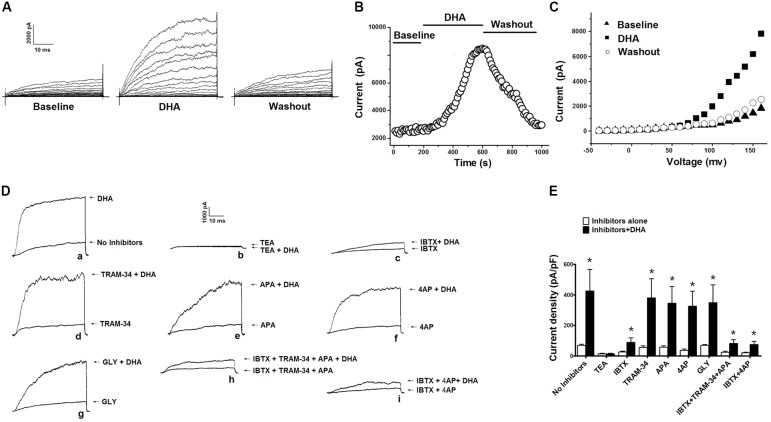
Effects of 5 μM DHA on total K^+^ currents in CASMCs. **(A)** Representative current traces showing outward K^+^ currents recorded from freshly isolated rat CASMCs at baseline, after exposure to 5 μM DHA, and after DHA washout. **(B)** Plot showing the time course of the experiment in **A**. **(C)** *I*–*V* relationship of total K^+^ currents at baseline, with application of 5 μM DHA, and after DHA washout. **(D)** Activation of total outward K^+^ currents in rat CASMCs by 5 μM DHA in the presence of various K^+^ channel blockers. Representative current traces showing the activation of total K^+^ currents in freshly isolated rat CASMCs by 5 μM DHA in the presence of various K^+^ channel blockers. **(E)** Group data in bar graphs, *n* = 5 cells, ^∗^*P* < 0.05 inhibitors alone vs. inhibitors + DHA.

In our previous study, we reported that BK channel activation in rat CASMCs by DHA at 1 μM was dependent on CYP epoxygenase activity (Wang et al., 2011). In this study, we found that activation of BK channels by DHA at >1 μM is independent of CYP epoxygenase activity. Total K^+^ currents were significantly increased by 5 μM DHA. Upon exposure to 5 μM DHA, the K^+^ currents were enhanced several folds from baseline, and these effects were reversed by DHA washout (**Figure 1A**). The time course of the effects of 5 μM DHA on K^+^ currents is shown in **Figure 1B**. The current–voltage (*I*–*V*) relationships of K^+^ currents at baseline, with DHA, and after washout are shown in **Figure 1C**.

Since there are different types of K^+^ channels in rat CASMCs, and the BK and Kv currents are the major constituents (Wang et al., 2011), we need to determine the components of K^+^ currents activated by 5 μM DHA in rat CASMCs. We examined the activation of DHA on K^+^ currents in the presence of various K^+^ channel blockers. With exposure to these blockers, total K^+^ currents were recorded before and after 5 μM DHA was applied, and the activation effects of DHA were determined by comparing the changes of K^+^ channel current densities. Representative current traces were shown in **Figure 1D** with the currents elicited from a HP of -60 mV and TP of +100 mV. The total K^+^ current densities before and after 5 μM DHA applied were 69.8 ± 6.9 and 425.0 ± 142.3 pA/pF (*n* = 5 cells, *P* < 0.05) with no inhibitors, 13.9 ± 2.7 and 14.1 ± 3.2 pA/pF (*n* = 5 cells, *P* > 0.05) in the presence of TEA (10 mM), 25.1 ± 5.6 and 89.1 ± 29.2 pA/pF (*n* = 5 cells, *P* < 0.05) in the presence of IBTX (100 nM), 55.8 ± 9.2 and 380.6 ± 125.5 pA/pF (*n* = 5 cells, *P* < 0.05) in the presence of TRAM-34 (200 nM), 56.5 ± 8.7 and 345.6 ± 110.1 pA/pF (*n* = 5 cells, *P* < 0.05) in the presence of 4AP (5 mM), 35.3 ± 9.9 and 326.6 ± 97.6 pA/pF (*n* = 5 cells, *P* < 0.05) in the presence of APA (1 μM), 68.6 ± 6.8 and 349.6 ± 115.9 pA/pF (*n* = 5 cells, *P* < 0.05) in the presence of GLY (10 μM), 23.0 ± 7.2 and 81.6 ± 23.7 pA/pF (*n* = 5 cells, *P* < 0.05) in the presence of IBTX (100 nM) plus TRAM-34 (200 nM) and APA (1 μM), and 20.9 ± 3.8 and 73.9 ± 21.9 pA/pF (*n* = 5 cells, *P* < 0.05) in the presence of IBTX (100 nM) plus 4AP (5 mM). The increasement of K^+^ currents was much smaller in the presence of IBTX than other blockers (*P* < 0.05), suggesting that BK currents are the major constituents of K^+^ currents activated by DHA in rat CASMCs. Group data are summarized in **Figure 1E**. However, after blocking K_Ca_ (in the presence of IBTX plus TRAM-34 and APA) or BK and Kv channels (in the presence of IBTX plus 4AP), there was still a small response to DHA, which may also activate chloride channels or other non-specific cation channels, such as transient receptor potential channels, etc.

### Effects of Different Concentrations of DHA on Whole-Cell BK Currents

**FIGURE 2:**
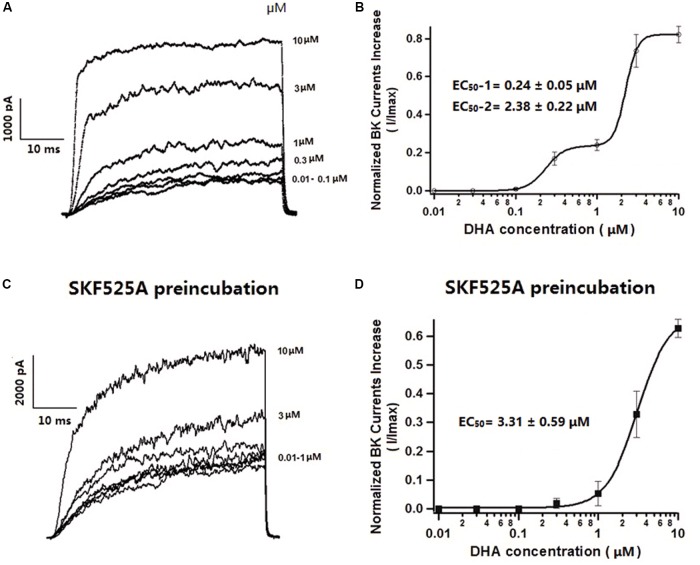
Effects of different concentrations of DHA on BK currents and the role of CYP in mediating DHA activation on BK currents in rat CASMCs. **(A)** Representative current traces showing BK currents at baseline and after exposure to 3 and 10 μM of DHA. **(B)** Concentration–response relationships showing the effects of DHA on BK channel activation, *n* = 6 cells. **(C)** Representative current traces showing BK currents at baseline and after exposure to 3 and 10 μM of DHA in rat CASMCs after incubation with 10 μM SKF525A for 60 min. **(D)** Concentration–response relationships showing the effects of DHA on BK channel activation with SKF525A pre-incubation, *n* = 6 cells.

Since BK channels are the major targets of DHA activation, further experiments were performed with 5 mM 4AP, 1 μM APA, and 200 nM TRAM-34 in the bath solution to block the Kv, SK, and IK channels. Under such conditions, BK currents were activated by DHA in a concentration-dependent manner. Representative current traces were displayed in **Figure 2A** (HP = -60 mV, TP = +100 mV). As we have previously reported (Wang et al., 2011), DHA had no effects at 0.01 and 0.03 μM, but activated BK currents at 0.1, 0.3, and 1 μM. Furthermore, 3 μM DHA increased BK currents by 737 ± 86% and 10 μM DHA increased BK currents by 822 ± 43% (*n* = 6 cells, *P* < 0.05). The DHA–BK current concentration–response curve is shown in **Figure 2B**. It is best described by curve fitting showing a double sigmoidal pattern with two plateaus (**Figure 2B**). Two half-maximal effective concentrations (EC_50_) are indicated: with a high potency of 0.24 ± 0.05 μM at low concentrations of DHA (0–1.0 μM) and a low potency EC_50_ of 2.38 ± 0.22 μM at high DHA concentrations (1.0–10 μM) (**Figure 2B**, *n* = 6 cells). This behavior of DHA on BK channel activation is unusual. These results suggested that DHA activates BK channels via different mechanisms at low and high concentrations.

### Effects of High Concentrations of DHA on Activation of Whole-Cell BK Currents Are Independent on CYP

We have previously reported that activation of BK channels by DHA at <1 μM is dependent on CYP epoxygenase activity and the activation effects of DHA on BK channels can be blocked by the CYP epoxygenase inhibitors SKF525A or MS-PPOH (Wang et al., 2011). To determine whether activation of BK channels by DHA at >1 μM is also dependent on CYP, freshly isolated rat CASMCs were pre-treated with 10 μM SKF525A for 60 min. Acute exposure to SKF525A (10 μM) had no effect on the IBTX-sensitive currents in CASMCs (Wang et al., 2011). After pre-treatment with SKF525A, baseline BK currents were the same as control group and DHA had no effects on BK currents at concentrations between 0.01 and 1 μM, but BK currents were increased by 327 ± 81 and 627 ± 32% at DHA concentrations of 3 and 10 μM, respectively (*n* = 6 cells, *P* < 0.05, **Figure 2C**). These findings suggested that activation of BK channels by DHA at low concentrations is dependent on CYP epoxygenase activity but not at higher concentrations. In the presence of SKF525A, DHA activated BK channels with an EC_50_ of 3.31 ± 0.59 μM (**Figure 2D**).

### Effects of Different Concentrations of DHA on Single BK Channel Currents

**FIGURE 3:**
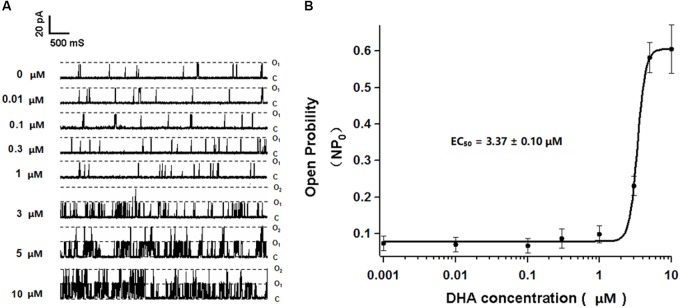
Effects of different concentrations of DHA on single BK currents in rat CASMCs. **(A)** Representative current traces showing single BK currents at baseline and after exposure to 0.01, 0.1, 0.3, 1, 3, 5, and 10 μM of DHA in rat CASMCs. **(B)** Concentration–response relationships showing the effects of DHA on BK channel activation, *n* = 5 cells.

Single BK channel currents were recorded from freshly isolated rat CASMCs in inside-out configuration. With 0, 0.01, 0.1, 0.3, and 1 μM DHA in the bath solution and in the presence of 1 μM free calcium, BK channel open probabilities at a membrane potential of +60 mV were 0.075 ± 0.019, 0.071 ± 0.020, 0.067 ± 0.019, 0.087 ± 0.026, and 0.098 ± 0.023, respectively (*n* = 5 cells, *P* > 0.05 vs. no DHA). In the presence of 3, 5, and 10 μM DHA, BK channel open probabilities were 0.232 ± 0.027, 0.582 ± 0.041, and 0.606 ± 0.066, respectively (*n* = 5 cells, *P* < 0.05 vs. no DHA). These results suggested that DHA had no direct effects on BK channels at concentrations of <1 μM but is a robust direct BK channel activator at >1 μM with an EC_50_ of 3.37 ± 0.10 μM (**Figures 3A,B**).

### Effects of DHA on Cytosolic Calcium Concentrations

**FIGURE 4:**
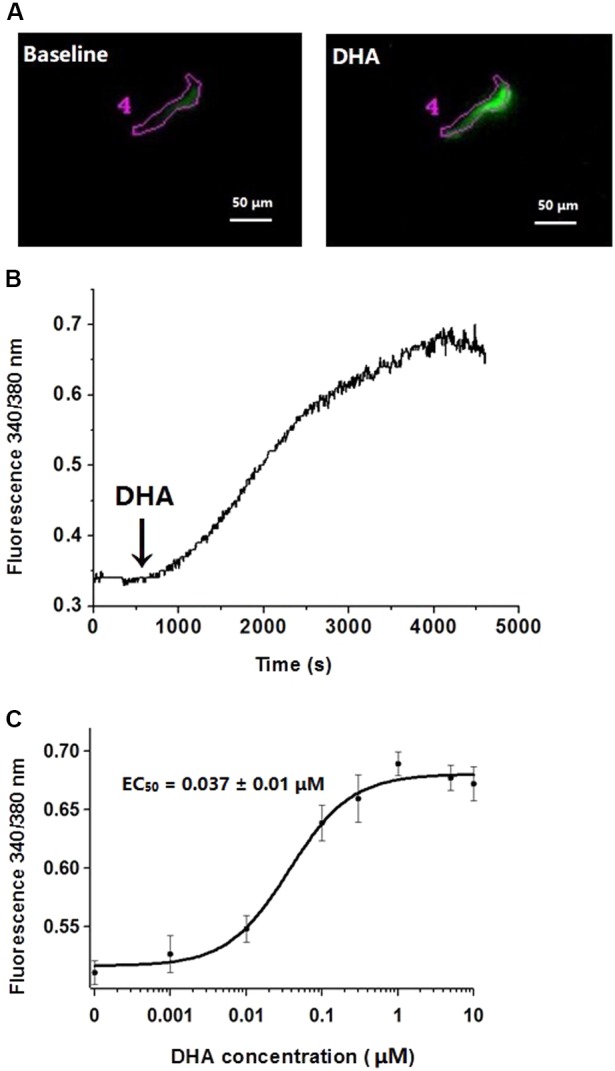
Effects of different concentrations of DHA on cytosolic calcium concentrations. **(A)** Representative changes of fluorescence intensity of F340 at baseline and after exposure 1 μM of DHA in rat CASMCs loaded with Fura-2/AM. **(B)** Representative changes of DHA-induced fluorescence intensity ratios. **(C)** Concentration–response relationships showing the effects of DHA on fluorescence intensity ratios, *n* = 5–8 cells.

It is well-known that BK channels in vascular smooth muscle cells are activated by an elevation of cytosolic calcium concentration. To determine whether DHA activates BK channels by elevated cytosolic calcium, we recorded signals from Ca^2+^-sensitive fluorescent indicators in the presence of different concentrations of DHA in rat CASMCs. In this experiment, one cell was used to investigate the effect of only one concentration DHA. DHA had no effects on cytosolic calcium concentrations ([Ca^2+^]*_i_*) at 0.001–0.01 μM, but fluorescence intensity ratios were increased on exposure to DHA at higher concentrations with an EC_50_ of 0.037 ± 0.01 μM (*n* = 5–8 cells for each concentration, *P* < 0.05) (**Figure 4**).

### DHA Induces the Increase in [Ca^2+^]*_i_* via PLC–IP_3_ Signal Pathway

**FIGURE 5:**
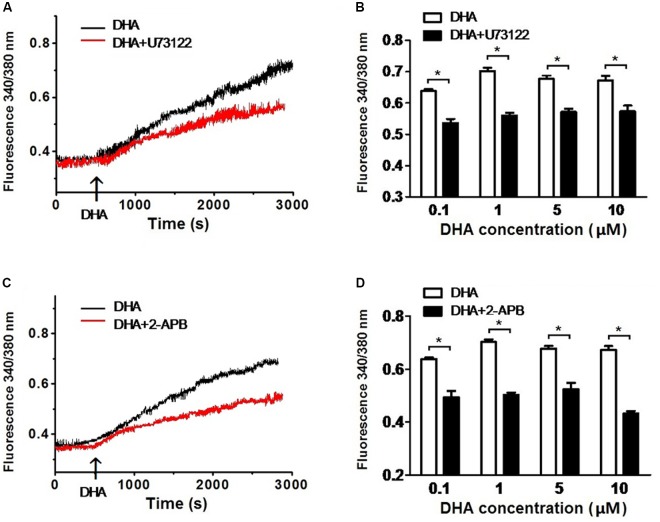
DHA induces the increase of [Ca^2+^]*_i_* via PLC–IP3 signal pathway. **(A)** Representative recordings of DHA-induced fluorescence intensity ratios in rat CASMCs with or without U-73122 pre-incubation. **(B)** Group data in bar graphs showing fluorescence intensity ratios after exposure to 0.1, 1, 5, and 10 μM of DHA in rat CASMCs with or without U-73122 pre-incubation, *n* = 5–8 cells, ^∗^*P* < 0.05 U-73122 vs. control. **(C)** Representative recordings of DHA-induced fluorescence intensity ratios in rat CASMCs with or without 2-APB pre-incubation. **(D)** Group data in bar graphs showing fluorescence intensity ratios after exposure to 0.1, 1, 5, and 10 μM of DHA in rat CASMCs with or without 2-APB pre-incubation, *n* = 5–8 cells, ^∗^*P* < 0.05 2-APB group vs. control group.

To determine the role of the PLC–IP_3_ pathway on the increase of [Ca^2+^]*_i_* by DHA, rat CASMCs were treated with U73122, an inhibitor of PLC and with 2-APB, a IP_3_ receptor blocker. After incubating with U73122, the fluorescence intensity ratios were significantly blunted upon exposure to DHA compared with controls (*n* = 5–8 cells, *P* < 0.05) (**Figures 5A,B**). Similarly, after incubating with 2-APB, the fluorescence intensity ratios were significantly blunted on exposure to DHA compared with controls (*n* = 5–8 cells, *P* < 0.05) (**Figures 5C,D**). These results indicated that DHA increases intracellular Ca^2+^ in rat CASMCs through PLC–IP_3_-dependent signal pathways. Hence, increase in intracellular Ca^2+^ is another potential mechanism through which DHA mediates the activation of coronary BK channels.

### Inhibition of PLC–IP_3_ Signal Pathway Reduces DHA-Induced BK Activation

**FIGURE 6:**
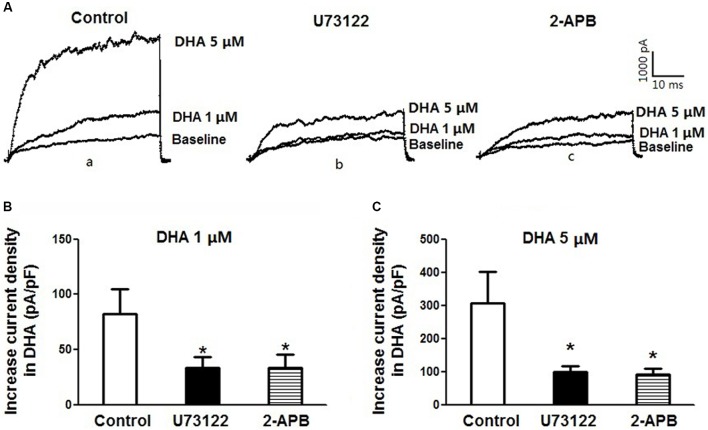
Inhibition of PLC–IP_3_ signal pathway reduces DHA-induced BK activation. **(A)** Representative current traces showing BK currents at baseline and after exposure to 1 and 5 μM of DHA of rat CASMCs in cells without treatment (control) **(a)** and those after incubation with U-73122 **(b)** or 2-APB **(c)**. **(B)** Group data in bar graphs showing increased current densities after exposure to 1 μM DHA in rat CASMCs with or without pre-incubation with U-73122 or 2-APB in bar graphs, *n* = 5 cells, ^∗^*P* < 0.05 c vs. control group. **(C)** Group data of increased current densities after exposure to 5 μM DHA of rat CASMCs with or without pre-incubation, *n* = 5 cells, ^∗^*P* < 0.05 vs. control.

To further confirm the role of PLC–IP_3_ signal pathway on BK activation by DHA, BK currents were recorded at baseline and after exposure to 1 and 5 μM of DHA in rat CASMCs (**Figure 6A,a-c**) with and without U73122 and 2-APB. Baseline BK current densities were not affected by incubation with U73122 and 2-APB (control: 68.3 ± 5.7 pA/pF; U73122: 66.1 ± 7.2 pA/pF; 2-APB 64.5 ± 3.0 pA/pF; *n* = 5 cells for each group; *P* > 0.05) but the ability of DHA at 1 and 5 μM in BK channel activation in CASMCs was significantly diminished (**Figures 6B,C**) (*n* = 5 cells for each group, *P* < 0.05). These results suggest that the PLC–IP_3_ signaling pathway is important in mediating BK channel activation by DHA in vascular smooth muscle.

## Discussion

In this study, we have made several important findings. First, DHA activates several K^+^ channels in CASMCs, but predominantly BK channels. Second, activation of BK channels by DHA has a double sigmoidal pattern with two plateaus. Low concentrations of DHA-induced BK channels activation are dependent on the activity of the CYP pathway, while high concentrations of DHA directly activate BK channels. Third, DHA increases the cytosolic calcium of rat CASMCs in a dose-dependent manner. Fourth, DHA can activate BK channels via the PLC–IP_3_–Ca^2+^ signal pathway. These important novel findings indicate that DHA activates the coronary BK channels through multiple mechanisms.

Large-conductance calcium-activated potassium channels are abundantly expressed in vascular smooth muscle cells (Wang et al., 2011), and their importance as a physiological regulator of vascular tone is well-recognized (Hoshi et al., 2013a). Cardiovascular diseases, such as hypertension (Nieves-Cintrón et al., 2007; Joseph et al., 2013) and diabetic vascular complications (Lu et al., 2010; Zhang et al., 2010; Yi et al., 2014), are associated with altered BK channel function in vascular smooth muscle cells. Hence, BK channel may be a potential therapeutic target for treatment of these diseases. We and other investigators have reported that DHA is a potent activator of vascular BK channels (Lu et al., 2010; Zhang et al., 2010; Wang et al., 2011). DHA produced dose-dependent vasodilatation of coronary vessels pre-constricted with endothelin-1. The effects were abolished in rat coronary vessels after incubated with IBTX, which indicates the vasodilatory effect of DHA is mediated through activation of BK channels (Wang et al., 2011). DHA could activate BK channels in rat CASMCs (Lai et al., 2009) and attenuate hypoxic pulmonary vasoconstriction in pulmonary artery smooth muscle cells (Yan et al., 2014). In contrast, the effects of lowering blood pressure by DHA were lost in BK channel knockout mice, suggesting that the effects of lowering blood pressure by DHA are mediated through the activation of BK channels (Lu et al., 2012). However, Sato et al. (2014) reported that the relaxant effects of DHA and its metabolites seemed to be partly triggered by the activation of K^+^ channels and the role for BK channels is insignificant. In this study, we found that DHA potently activated total K^+^ currents but this effect was very weak in the presence of IBTX, a specific BK channel blocker, indicating that the activation of K^+^ currents in CASMCs by DHA is mainly mediated through BK channels.

We have previously reported that the effects of DHA at <1 μM on BK channel activation were dependent on CYP epoxygenase activity and abolished by pre-incubation with a CYP inhibitor (SKF525A or MS-PPOH) in rat CASMCs (Wang et al., 2011). Recently, BK channel activation by the CYP products of DHA has been extensively studied (Ye et al., 2002; Lucas et al., 2010; Agbor et al., 2012). There are six epoxydocosapentaenoic acid (EpDPE) regioisomers (4-, 5-, 7-, 8-, 10-, 11-, 13-, 14-,16-, 17-, 19-, and 20-EpDPE), which are CYP-dependent epoxygenase metabolites of DHA (Lucas et al., 2010). The CYP knockout mice exhibited a significantly attenuated vasorelaxation response to DHA, but a normal vasorelaxation response to the CYP metabolites, suggesting that CYP metabolizes DHA to vasodilators *in vivo* (Agbor et al., 2012). Ye et al. (2002) also reported that 13-, 14-EpDPE activated BK channels in porcine coronary arterioles and rat small coronary arteries. Indeed, we also reported that 16-, 17-EpDPE activated BK whole-cell currents in a dose-dependent manner (Wang et al., 2011).

Since human plasma concentrations of DHA is in the micromolar range (Harper et al., 2006), we found that micromolar concentrations of DHA directly activate BK channels independent of CYP epoxygenase activity. DHA activates BK channels in a bimodal manner with two EC_50_ as shown in **Figure 2**. The effects of DHA epoxides are potent activators of BK channels but their effects appear to be saturated at sub-micromolar concentrations. Hence, the dietary consumption of DHA represents an important source of BK channel activators help preserve cardiovascular function including blood pressure control and vital tissue perfusion.

A recent study suggested that the activation of BK channels by 3 μM DHA is not dependent on the activation of the voltage or Ca^2+^ sensors of BK channels (Hoshi et al., 2013d). DHA directly acts on BK channels through a single residue Y318 at the C-terminus of S6 segment in the pore-gate domain of the α-subunit. However, when DHA was applied to heterogeneously expressed channels containing only the pore-forming α-subunit, the activation effects of DHA were very limited (Hoshi et al., 2013c), and the stimulatory effects of DHA were particularly noticeable when the α-subunit was co-expressed with the auxiliary β1-subunit in vascular cells (Knaus et al., 1994; Wallner et al., 1995) or with β4 in neurons (Meera et al., 2000). Hence, the presence of β1- or β4-subunit potentiates the functional consequence of DHA binding to Y318 at the S6 segment in α-subunit.

We have previously reported that DHA enhanced the activities of spontaneous transient outward currents (STOCs) in CASMCs (Wang et al., 2011). The STOCs are sensitive to IBTX and can be used as measurements of BK channel activation by local transient increase in intracellular Ca^2+^ (Wang et al., 2011). In human (Jurkat) T-cell lines, DHA evoked an increase in [Ca^2+^]*_i_* which was not observed during the anti-CD3-induced calcium peak though its addition resulted in a prolonged and sustained calcium response. DHA-induced sustained response on the increases in [Ca^2+^]*_i_* was also curtailed after pre-incubation of T cells with tyrphostin A9, an inhibitor of Ca^2+^ release-activated Ca^2+^ (CRAC) channels (Bonin and Khan, 2000). Thus, DHA induces an increase in [Ca^2+^]*_i_* via the ER pool and the opening of CRAC channels in human T cells. DHA can also induce a dose-dependent increase in [Ca^2+^]*_i_* through activation of PLC–IP_3_ pathway and activation of PKCγ/δ, which may be involved in apoptosis of monocytic leukemia U937 cells (Aires et al., 2007). In this study, we found that DHA produced a dose-dependent increase in [Ca^2+^]*_i_* in rat CASMCs and this effect was attenuated by pre-incubation with U73122, an inhibitor of PLC or 2-APB, an IP3 blocker, suggesting that the DHA induced-increase in intracellular Ca^2+^ is mediated through the PLC–IP_3_ mechanism. BK channels were highly sensitive to Ca^2+^ and the activation of BK currents required an elevation of intracellular Ca^2+^. Several studies have reported the PLC–IP_3_–[Ca^2+^]*_i_* signal pathway involved in BK channels activation. In coronary smooth muscle cells, adenosine triphosphate may elevate [Ca^2+^]*_i_* via purine receptor (P2Y1)–PLC–IP_3_ pathway consequently activating BK channels (Wang et al., 2015). The oxytocin hyperpolarized myenteric intrinsic primary afferent neurons by activating BK channels via the oxytocin receptor – PLC–IP_3_–Ca^2+^ signal pathway. And the effect of oxytocin on the BK currents was blocked by pre-treatment with 2-APB or U73122 (Che et al., 2012). DHA dilated resistance pulmonary arteries and the effects of DHA were abolished after pre-treatment with IP_3_ receptor inhibitor or IBTX, suggesting a role of IP_3_ in BK channels activation by DHA (Yan et al., 2014). To further confirm the relationship between PLC–IP_3_ revelated [Ca^2+^]*_i_* and BK channel activation by DHA, the PLC–IP_3_–[Ca^2+^]*_i_* signal pathway was blocked, and we found that it attenuated BK activation in CASMCs.

**FIGURE 7:**
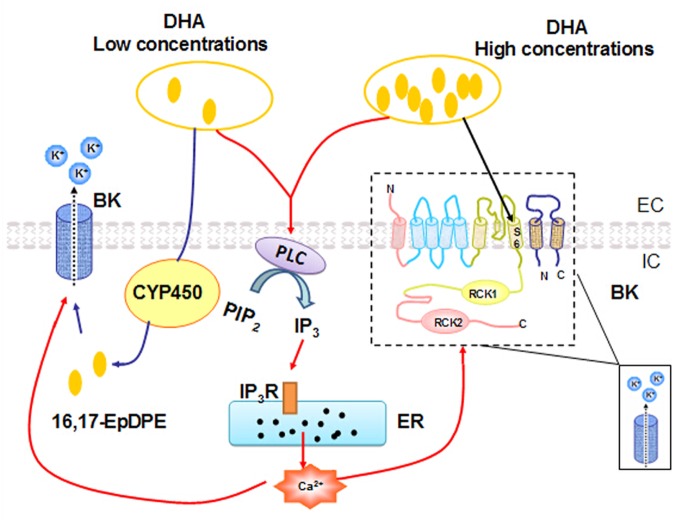
A proposed model for BK channel activation mechanisms by different concentrations of DHA. Low concentrations of DHA-induced BK channel activation are dependent on CYP epoxygenase activity. High concentrations of DHA activate BK channels directly. In addition, DHA can also induce a dose-dependent increase in [Ca^2+^]*_i_* via the PLC–IP_3_–Ca^2+^ signal pathway, and the ensuing increased [Ca^2+^]*_i_* can activate BK channels in rat CASMC.

To summarize, our results demonstrated that DHA can activate BK channels through multiple mechanisms (**Figure 7**). DHA-induced BK channel activation at low concentrations (<1 μM) is mediated through CYP epoxygenase metabolites, while DHA at high concentrations (>1 μM) directly activates BK channels, probably by binding to the S6 segment in the α-subunit. In addition, DHA can activate BK channels through elevated cytosolic calcium via the PLC–IP_3_–Ca^2+^ mechanism. BK channel should be considered as a novel therapeutic target for the prevention and/or treatment of vascular abnormalities in diabetes, hypertension, and other vasculopathic conditions.

## Author Contributions

R-XW, L-LQ, and M-QS were involved in the experiment design. L-LQ, M-QS, R-XW, TL, YW, YJ, XT, X-XZ, and WW performed the experiments. L-LQ, S-PD, X-YL, and R-XW analyzed the data. L-LQ and M-QS wrote the manuscript. R-XW, TL, H-CL, QC, MP, FY, and D-MZ edited the manuscript. All authors read and approved the final manuscript.

## Conflict of Interest Statement

The authors declare that the research was conducted in the absence of any commercial or financial relationships that could be construed as a potential conflict of interest.
